# Insights on Minimizing False Positives in IHHNV Detection: Experiences from Ecuador’s *Penaeus vannamei* Aquaculture

**DOI:** 10.3390/ijms262311484

**Published:** 2025-11-27

**Authors:** Pablo Intriago, Melany del Barco, María Mercedes Vásquez, Bolivar Montiel, Ronald Villamar

**Affiliations:** 1 South Florida Farming Corp, 13811 Old Sheridan St, Southwest Ranches, FL 33330, USA; 2 South Florida Farming Lab, Av. Miguel Yunez, Km 14.5 via a Samborondón, Almax 3 Etapa 1-Lote 3 Bodega 2, Samborondón, Guayas, Ecuador

**Keywords:** *Penaeus vannamei* (*Pv*), *Penaeus monodon* (*Pm*), infectious hypodermal and hematopoietic necrosis virus, renamed Penstylhamaparvovirus 1 (IHHNV), runt deformity syndrome (RDS), World Organization for Animal Health (WOAH), endogenous viral elements (EVEs)

## Abstract

Detection of infectious hypodermal and hematopoietic necrosis virus (IHHNV) in shrimp aquaculture is complicated by endogenous viral elements (EVEs) causing false positives in conventional PCR assays. This study analyzed 277 *Penaeus vannamei* samples from Ecuador using World Organization for Animal Health (WOAH)-recommended short-fragment primers (IHHNV-309, -389, -392, -77012; ~1.5 kb amplicons) and long-amplicon PCR (LA-PCR; ~3.7 kb, 90% of the genome), complemented by histopathology. Short-fragment primers showed high positivity rates (72.9–83.0% individually; 69.3% combined), while LA-PCR reduced positives to 29.6%, with 95.1% overlap indicating true infections as a subset of conventional results. Approximately 55.6% of samples likely contained EVEs mimicking IHHNV, and 14.8% were true negatives. Histopathology confirmed classic IHHNV lesions (Cowdry A-type inclusions) in only one sample (0.36%), which also showed co-infections (hepatopancreatic atrophy, gregarines, and unidentified viral inclusions), suggesting multifactorial pathology. These findings highlight inflated IHHNV prevalence due to single-primer PCR, particularly in Ecuador, where reliance on WOAH-suggested primers (e.g., 389F/R) fails to distinguish infectious IHHNV from EVEs or confirm subclinical status, risking misattribution of losses to IHHNV while overlooking pathogens like *Vibrio* spp. We advocate LA-PCR and histopathology to enhance diagnostics and support sustainable shrimp fisheries.

## 1. Introduction

### 1.1. General Background

Infectious hypodermal and hematopoietic necrosis virus (IHHNV), now classified as *Penstylhamaparvovirus* 1 [1], is a parvovirus with a complex role in shrimp aquaculture. Historically a significant pathogen, its current status as an active infectious agent versus endogenous viral elements (EVEs) remains uncertain, complicating accurate diagnosis. Despite its diminished impact on global shrimp production, IHHNV continues to challenge diagnostic reliability. For instance, Sritunyalucksana et al. [2] reported no significant impact on *Penaeus monodon* production in Thailand since 2010, highlighting limitations in relying solely on polymerase chain reaction (PCR) assays for detection.

In their investigation of 11 commercial ponds, the World Organization for Animal Health (WOAH)-recommended PCR methods (IHHNV-309 and IHHNV-389) produced false-positive results in 82% of cases, attributable to EVEs integrated into the shrimp genome [3]. The combination of these four traditional IHHNV primers collectively amplifies only approximately 1.5 kb of the IHHNV genome [4,5]. In contrast, an in-house long-amplicon PCR method (IHHNV-LA) [5], which amplifies approximately 90% (ca. 3.7 kb) of the 4 kb IHHNV genome, minimizes false positives; when integrated with histological examination, it confirmed active infections in only two ponds, with no pathognomonic Cowdry A-type intranuclear inclusions or production losses observed in the majority. These results highlight the necessity for multifaceted diagnostic strategies, incorporating long-amplicon PCR with histopathology, to achieve precise IHHNV evaluations and promote sustainable shrimp farming practices.

### 1.2. IHHNV Characteristics and Historical Impact

IHHNV, classified as *Penstylhamaparvovirus* 1 (family Parvoviridae, subfamily Hamaparvovirinae), is a small (20–22 nm diameter), non-enveloped, icosahedral virus containing a linear, single-stranded DNA genome of approximately 4.1 kb [1,2]. It was initially identified in post-larvae of *P. stylirostris* and *P. vannamei* from hatcheries in Costa Rica, Ecuador, Florida, and Tahiti [6,7]. IHHNV infects all life stages of penaeid shrimp, primarily targeting ectodermal and mesodermal tissues, including the gills, cuticular epithelium, connective tissues, hematopoietic tissues, lymphoid organs, antennal glands, and nervous system [3]. Recent research by Arbon et al. [8] investigated how IHHNV distributes in different tissues of giant black tiger shrimp (*P. monodon*). The study found that the highest viral load occurs in the gills, with an average of approximately 10^7^ viral copies per milligram of tissue. Other tissues showed progressively lower viral loads in the following order: hindgut, pleopod, hepatopancreas, lymphoid organ, ventral nerve cord, and tail, with the tail having the lowest viral load. The hindgut had a high viral load, but results should be interpreted cautiously due to possible contamination from non-viable viral DNA in feed.

In *P. stylirostris*, acute infections lead to high mortality rates, whereas in *P. vannamei*, chronic infections manifest as runt deformity syndrome (RDS) [7,9], characterized by stunted growth, bent rostra, abdominal deformities, wrinkled antennae, and irregular cuticles [10].

In 1992, Owens et al. [11] reported Australia’s first confirmed epizootic of IHHNV, resulting in 100% mortality in hybrid prawns (*P. esculentus* × *P. monodon*). The outbreak involved a novel IHHNV genetic variant, with Cowdry type A inclusions (CAIs) widespread in ectodermal and mesodermal tissues, signaling severe infection. Co-infections with Lymphoidal parvovirus (LPV) and Plebejus baculovirus (PBV) likely amplified the susceptibility of these hybrid prawns, driving the extreme mortality. In contrast, Krabsetsve et al. [12], years later, identified IHHNV as an endemic, ancient lineage in Australia, causing no significant mortality in wild *P. monodon*. This suggests the 1992 epizootic’s severity stemmed from the hybrid prawns’ genetic makeup and co-infections rather than IHHNV alone.

More recently, Sellars et al. [13] documented the first yield reduction in farmed *P. monodon* in Australia linked to high-load IHHNV, reporting vertical transmission and reduced growth rates that caused significant economic losses without runt deformity syndrome (RDS). However, their PCR-based analysis, which only tested for IHHNV and gill-associated virus/yellow head virus 7 (GAV/YHV7), failed to distinguish infectious IHHNV from endogenous viral elements (EVEs), first identified by Tang et al. [14], and did not screen for other pathogens like *Enterocytozoon hepatopenaei* (EHP), *Vibrio* spp., or monodon baculovirus (MBV) or incorporate histopathology. This leaves IHHNV’s role in the losses unconfirmed, as environmental factors such as low oxygen, pH fluctuations, or high ammonia could also explain the growth reduction. In contrast, Ref. [15] developed real-time PCR assays with high sensitivity (detecting ≤10 DNA copies) to differentiate infectious IHHNV lineages (I, II, III) from non-infectious EVEs in *P. monodon* from Australia and Vietnam/Malaysia, enabling reliable broodstock screening [15]. These assays address the diagnostic limitations in Sellars et al.’s study, offering a tool to clarify whether infectious IHHNV, rather than EVEs or other factors, drove the observed economic impacts, underscoring the need for integrated diagnostics in *P. monodon* aquaculture.

In Ecuador, Jiménez et al. [16] documented elevated IHHNV prevalence in farmed *P. vannamei* during February to May 1996, exacerbated by El Niño-induced environmental stressors (salinity < 5 ppt, temperatures > 29 °C) and high stocking densities. This resulted in significant growth retardation (juvenile weights of 0.09–3.0 g versus expected 8–12 g at harvest) without associated mortality or overt RDS symptoms, contributing to substantial economic losses by 1998. No significant IHHNV-related impacts have been reported in Ecuador or other Latin American regions for over 25 years since this report.

Histopathologically, IHHNV induces pathognomonic Cowdry A-type intranuclear inclusions in affected tissues of both *P. vannamei* and *P. stylirostris*, including the cuticular epithelium, gills, nerve cord, antennal gland, and hematopoietic tissue [16]. Infected cells exhibit chromatin margination, nuclear clearing, hypertrophy, and eosinophilic or basophilic inclusions. Ultrastructural examination reveals cytoplasmic virogenic stroma and viral aggregates, occasionally arranged in paracrystalline arrays [16]. Notably, inclusion bodies are less abundant in larger shrimp, indicating diminished disease severity with increasing host size [16]. These tissue-specific histopathological features are essential for precise diagnosis and elucidating the variable clinical manifestations of IHHNV infection.

### 1.3. Diagnostic Challenges and Genotypic Variability

IHHNV, listed in the WOAH Aquatic Animal Health Code since 1995, has three genotypes: Types 1 and 2 (infectious) and Type 3 (non-infectious EVEs integrated into the *P. monodon* genome) [15,17]. Conventional PCR (309F/R for infectious IHHNV, 389F/R for all genotypes) often misidentifies EVEs as active infections, with 389F/R amplifying non-infectious sequences in *P. monodon* from Australia, Africa, and the Western Indo-Pacific [18,19]. Recent studies confirm multiple EVE clusters in *P. monodon*, complicating short-fragment PCR reliability [20]. WOAH’s alternative primers (77013/77353, 392F/R) are outdated [3,18,21], and a confirmed IHHNV infection requires at least two of the following: positive in situ hybridization (ISH), genotype-specific PCR, or sequence analysis [22]. The IHHNV-LA method, combined with histopathology, offers superior diagnosis by distinguishing single-stranded viral DNA from double-stranded EVEs.

#### Ecuadorian Industry Resilience and Implications for SPF Certification and Trade

Since 2010, IHHNV has had minimal impact on Ecuador’s *P. vannamei* shrimp industry, as evidenced by export growth from 180,000 MT to 1,250,000 MT by 2023, reflecting almost a 15% annual increase [23]. From 1999 to 2010, white spot syndrome virus (WSSV) outbreaks caused severe mortality, masking IHHNV and co-pathogens like *Vibrio* spp., complicating their assessment [16]. Since 2001, mass selection for WSSV resistance, driven by high losses and a ban on wild post-larvae, combined with advanced pond management (improved pumping, aeration, automatic feeders, and high-quality feeds), has transformed Ecuador into the world’s leading *P. vannamei* exporter. Surveillance studies in 2023–2024, examining over 300 animals across the region, found no Cowdry A-type inclusions despite frequent *Vibrio* co-infections, confirming low active IHHNV infection and no RDS [23,24]. High WOAH PCR positivity (13/15 ponds) reflects false positives from EVEs, not active infection [25].

The Ecuadorian shrimp industry and regulatory authorities heavily rely on single-primer PCR testing, using WOAH-suggested primers, to detect IHHNV and WSSV as primary causes of hatchery and farm outbreaks, despite their distinct pathologies complicating accurate diagnosis. While WSSV PCR results might indicate the presence of a virulent pathogen that could result in an outbreak, IHHNV testing with single primers (e.g., 389F/R) fails to distinguish between infectious virus and non-infectious endogenous viral elements (EVEs), nor does it clarify subclinical status, potentially misattributing losses to IHHNV. This approach overlooks other pathogens, especially *Vibrio* spp., and hinders the adoption of comprehensive diagnostics, such as multi-pathogen screening or histopathology, needed for precise outbreak management [23,24].

This overemphasis not only disrupts trade through unjustified export and import barriers but also hampers local sales, including post-larvae transactions from hatcheries, while representing a substantial waste of energy and funds on a virus that, as evidenced by Ecuador’s robust shrimp export growth, is no longer a significant pathogen. Given the minimal impact of IHHNV on modern shrimp production, it should be considered for delisting from the WOAH list of reportable aquatic diseases to align regulations with current scientific evidence and reduce unnecessary burdens on the industry [22]. These findings underscore Ecuador’s resilience and position it as a paradigm for global shrimp aquaculture, advocating the adoption of integrated diagnostic approaches, such as IHHNV long-amplicon PCR [4] combined with histopathology, to mitigate EVE-induced false positives and foster sustainable production [22].

Endogenous viral elements (EVEs) pose significant challenges to specific pathogen-free (SPF) certification in shrimp aquaculture, as they frequently trigger false-positive results in World Organization for Animal Health (WOAH) polymerase chain reaction (PCR) assays, potentially leading to unjustified trade restrictions not only for SPF-certified stocks [14,26] but also for non-SPF populations, such as those from Ecuador, where no IHHNV-related impacts have been observed over the past two decades despite occasional PCR positives. Genetic recombination among EVE fragments can generate “pop-up” false positives, exacerbating certification complexities [27]. Analogous issues have been documented with white spot syndrome virus (WSSV) EVEs in *P. monodon*. In Latin America, particularly Ecuador, non-SPF populations of *P. vannamei* have exhibited no discernible IHHNV-associated adverse effects or RDS over the past two decades, indicating potential host tolerance or minimal pathogen impact under prevailing environmental and management conditions. In contrast, SPF *P. vannamei* stocks demonstrate superior growth rates while remaining free of IHHNV and other major pathogens [2].

## 2. Results

### 2.1. PCR Results

A total of 277 samples were analyzed using the conventional primers IHHNV-309, IHHNV-389, IHHNV-392, and IHHNV-77012, as well as the long-amplicon PCR (LA-PCR) method. Table S1 presents the results for all primer pairs, with positivity rates decreasing from IHHNV-309 (230/277; 83.0%) to IHHNV-389 (229/277; 82.7%), IHHNV-392 (224/277; 80.9%), and IHHNV-77012 (202/277; 72.9%). Overall, 192/277 samples (69.3%) tested positive for all four conventional primer pairs. In contrast, when compared to the LA-PCR method, the positivity rate dropped markedly to 82/277 samples (29.6%). Notably, 78 of these 82 LA-PCR-positive samples were also positive for all four conventional primer pairs. Additionally, 41/277 samples (14.8%) tested negative for all primer pairs.

### 2.2. Histopathology Results

**Figure 1:**
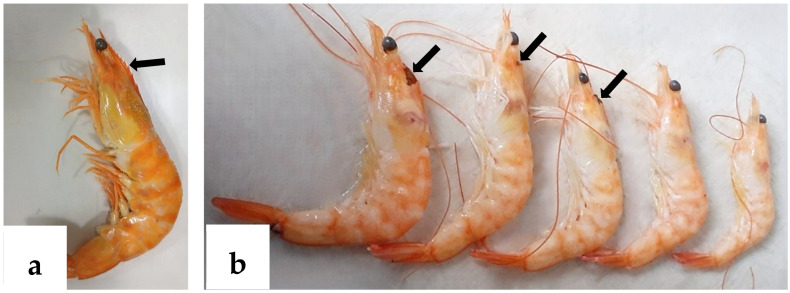
*P. vannamei* specimens fixed in Davidson’s solution. (**a**) Morphologically normal shrimp with intact rostrum (arrow). (**b**) Shrimp exhibiting size variation and melanized rostrum deformities (arrows).

Histological examination of 277 *P. vannamei* samples fixed in Davidson’s solution revealed classic IHHNV lesions in one sample (0.36%), displaying a deformed, melanized rostrum with size variation, marked hemocytic infiltration, pyknotic nuclei, and pathognomonic Cowdry type A intranuclear inclusion bodies (CAIs) in the cuticular epidermis, contrasting with morphologically normal shrimp with intact rostrums (Figure 1a,b).

**Figure 2:**
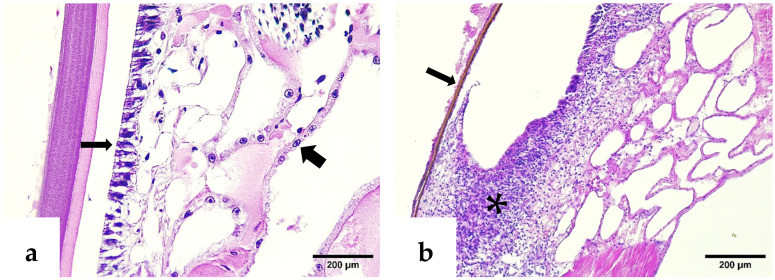
H&E–phloxine-stained sections of *P. vannamei* rostrums. (**a**) Normal rostrum with intact epidermal and hypodermal layers (thin arrow) and antennal gland epithelial cells (thick arrow). (**b**) IHHNV-infected rostrum exhibiting cuticular melanization (thin arrow) and marked inflammatory response in hypodermal tissue (asterisk).

**Figure 3:**
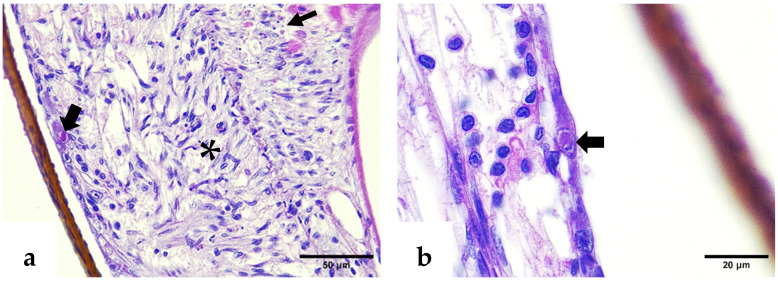
H&E–phloxine-stained sections. (**a**) Closer view of a deformed rostrum; note the affected hypodermis (asterisk) with clusters of pyknotic nuclei (thin arrow), and a Cowdry type A inclusion body of IHHNV within the epidermis (thick arrow). (**b**) Pathognomonic CAI of IHHNV infection located in the epidermis of another deformed rostrum (thick arrow).

H&E–phloxine-stained sections showed a deformed rostrum with cuticular melanization, marked hemocytic infiltration, pyknotic nuclei, and pathognomonic Cowdry type A intranuclear inclusion bodies (CAIs) in the epidermal layers. In contrast, normal rostrums exhibited intact epidermal, hypodermal, and antennal gland epithelial structures (Figure 2 and Figure 3).

**Figure 4:**
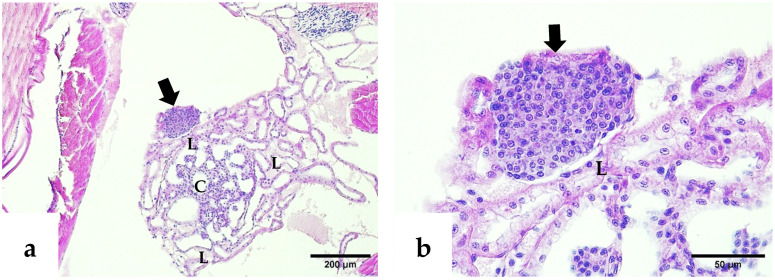
H&E–phloxine-stained sections. (**a**) Ectopic spheroid located above the labyrinth of the compact glandular compartment (thick arrow). (**b**) Higher magnification of the compact glandular compartment revealing the ectopic spheroid positioned over the labyrinthine cells (thick arrow). C: coelomosac lumen; L: labyrinth.

**Figure 5:**
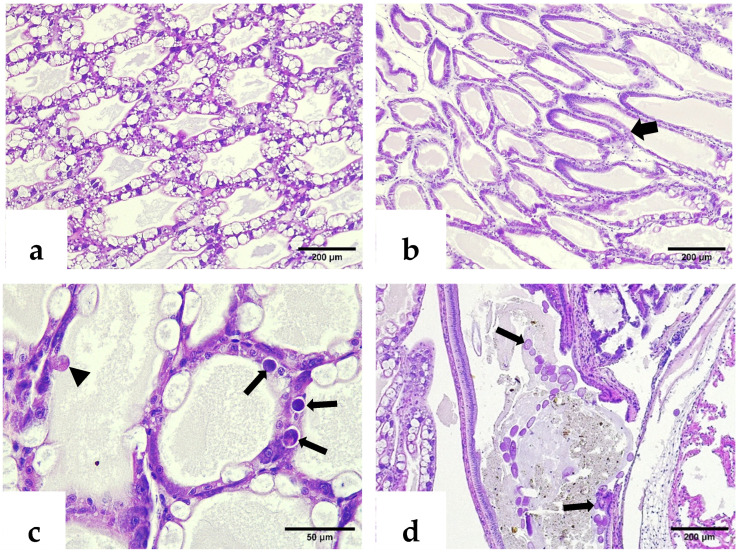
(**a**) H&E–phloxine-stained sections. (**a**) General view of a hepatopancreas with normal appearance and abundant storage bodies. (**b**) Hepatopancreatic tubules from an affected shrimp showing marked atrophy and absence of storage bodies (thick arrow). (**c**) A closer view reveals early cellular detachment (arrowhead) and basophilic inclusion bodies (thin arrows). (**d**) Infestation of gregarines within the affected midgut, observed both attached to the epithelium and free in the lumen (thin arrows).

Histological examination also revealed ectopic spheroids in the compact glandular compartment of the nephrocomplex (Figure 4a,b). The hepatopancreatic epithelium showed atrophy by intraluminal distension, absence of storage bodies, cellular detachment, and cytoplasmic inclusion bodies suggestive of Wenzhou shrimp virus 8 (WzSV8) (Figure 5a,c). Gregarines at various developmental stages, both attached to the intestinal epithelium and free in the lumen, were associated with peritrophic membrane disruption and smooth muscle alterations (Figure 5d).

**Figure 6:**
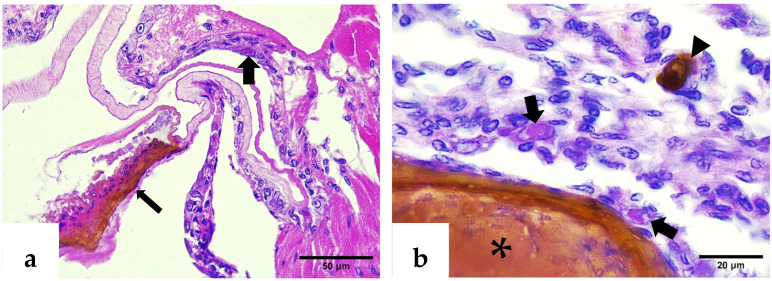
H&E–phloxine-stained sections. (**a**) Deformed pleopod with melanized cuticle (thin arrow) and intranuclear IHHNV CAI in the epidermis (thick arrow). (**b**) Affected abdominal segment; note the melanosis in the cuticle (asterisk), the melanin deposit in the hypodermal tissue (arrowhead), and eosinophilic intranuclear IHHNV CAIs (thick arrows).

Similar to the lesions observed in deformed rostrums, intranuclear eosinophilic inclusion bodies associated with IHHNV were detected in the epithelial cells of affected pleopods and abdominal segments (Figure 6a,b). These CAIs were consistently accompanied by melanized reactions in the cuticle and significant structural alterations in epithelial cells. Collectively, these histopathological changes suggest a strong association between the moderate IHHNV infection and a localized tissue response.

**Figure 7:**
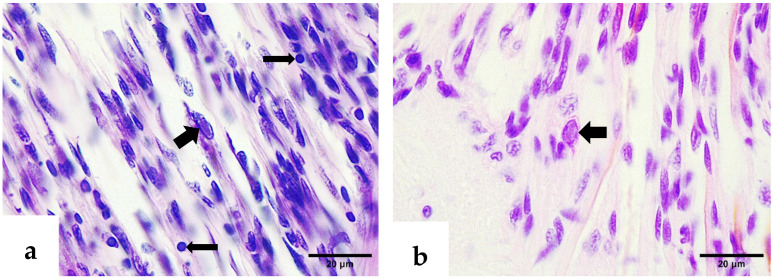
(**a**) H&E–phloxine-stained section through the ventral nerve cord showing an intranuclear elongated inclusion body of IHHNV (thick arrow), while multiple pyknotic nuclei are also evident (thin arrows). (**b**) Methyl green–pyronin-stained section through the circumesophageal connectives, highlighting an IHHNV-infected cell (CAI) with pinkish intranuclear coloration (thick arrow).

Examination of the ventral nerve cord revealed CAIs with atypical morphologies, likely due to their conformation to the shape of host cell nuclei (Figure 7a). The presence of pyknotic nuclei suggests a degenerative response to the viral infection. Methyl green–pyronin staining highlighted a characteristic IHHNV CAI in the circumesophageal connectives, paired neural tracts connecting the supraesophageal ganglion to the ventral nerve cord, displaying a pinkish hue (Figure 7b). This coloration likely reflects the acidophilic nature of the inclusion, which may vary with the stage of virogenesis.

## 3. Discussion

This study demonstrates that the conventional primers (IHHNV-309, -389, -392, and -77012) yielded high individual positivity rates, ranging from 83.0% to 72.9%, with 69.3% of samples positive across all four. Collectively, these primers amplify approximately only 1.5 kb of the 4 kb IHHNV genome [5], rendering them susceptible to detecting incomplete or integrated sequences like endogenous viral elements (EVEs) rather than full infectious viral genomes [2,3,20,28,29]. This suggests that routine screening with these primers alone likely inflates the perceived prevalence of active IHHNV infections, as they cannot reliably distinguish between true viral replication and non-infectious genomic integrations. In contrast, the long-amplicon PCR (LA-PCR) method, which amplifies approximately 90% (3.7 kb) of the IHHNV genome [5], detected positives in only 29.6% of samples, a dramatic reduction compared to the conventional methods. This approach minimizes false positives by requiring a near-complete genome for amplification, effectively filtering out fragmented EVEs. Notably, nearly all LA-PCR positives (78/82, or 95.1%) overlapped with samples positive for all four conventional primers, indicating that true infections represent a subset of conventional positives, while most of the latter are artifacts. At least 55.6% of samples (those positive in at least one conventional assay but negative in LA-PCR) likely contain EVEs mimicking IHHNV sequences, rather than active virus. Additionally, 14.8% of samples were negative across all methods, suggesting they are truly IHHNV-free (no virus or EVEs detected). This implies that EVEs are prevalent in the tested population (potentially up to ~70% if considering partial conventional positives), but they do not represent infectious threats, aligning with observations in species like *P. monodon* and *P. vannamei*, where EVEs are integrated into the host genome without causing disease. Supporting this, histological examination of the 277 samples revealed that only one displayed classic histopathological lesions pathognomonic of IHHNV (e.g., Cowdry type A intranuclear inclusion bodies), further underscoring the rarity of active infections. Notably, this single sample also exhibited co-infections characteristic of regionally reared *P. vannamei* [23,24], including hepatopancreatic atrophy with intraluminal distension, cellular detachment, inclusion bodies (WzSV8), and gregarine parasites at various stages disrupting the peritrophic membrane and smooth muscle layer, suggesting that any observed pathology may be multifactorial rather than solely attributable to IHHNV typically found in the region [23,24].

The discrepancy between methods underscores the need to shift from short-fragment PCR (e.g., WOAH-recommended primers) to more robust approaches like long-amplicon PCR (LA-PCR), especially in regions with historical IHHNV exposure where endogenous viral elements (EVEs) may be prevalent. Over-reliance on conventional primers could impose unnecessary economic burdens, such as trade restrictions, culling, or misattribution of production issues to IHHNV when other factors are responsible. In contexts like Ecuador’s resilient shrimp industry, where IHHNV impacts have been minimal over the past 15 years despite high conventional PCR positives, this supports delisting IHHNV from reportable disease lists if EVEs are the primary driver of detections. Overall, true active IHHNV infections appear low (≤29.6%), and the data advocate for integrated diagnostics (e.g., LA-PCR combined with histopathology) to ensure accurate assessments and promote sustainable production.

Caicedo et al. [30] described the diagnosis and reporting of IHHNV infection in two shrimp farms on Colombia’s north coast in 2021. Epidemiological investigation revealed IHHNV prevalence of 97% and 36% in the two farms using WOAH-recommended PCR. Histopathology showed no inclusion bodies, but in situ hybridization (ISH) yielded positives interpreted as confirmation of infection. However, if EVEs—non-infectious fragments of IHHNV integrated into the shrimp’s chromosomal DNA—contain those same sequences, the probe can bind to them, producing a positive signal even in the absence of replicating virus. EVEs are inherited and present in every cell of the host, so the ISH signal might appear more diffuse or ubiquitous (e.g., in all nuclei) rather than localized to infected tissues like the hypodermis or hematopoietic organs. In [30], ISH was positive, but histopathology showed no Cowdry A-type inclusion bodies (the hallmark of active IHHNV replication and disease). This mirrors our findings and suggests the ISH signal may stem from EVEs rather than the infectious virus, as active IHHNV typically induces visible cellular changes (e.g., nuclear hypertrophy and inclusions) in ectodermal/mesodermal tissues. Without pathology or clinical signs (e.g., runt deformity syndrome), a positive ISH alone is insufficient to rule out EVEs. This EVE-driven ambiguity is further supported by Zhong et al. [31], who analyzed 105 crustacean genomes and identified 252 IHHNV-EVEs (183 ancient, 6 recent), distributed across Decapoda, Thoracica, and Isopoda. Their work highlights EVE expansion in Decapoda as evidence of an ongoing host–virus arms race, with integrations occurring only in susceptible species exploiting host DNA replication machinery. While most recent EVEs were laboratory contaminants, one intact integration in *P. monodon* persisted across populations, underscoring EVEs as dynamic “molecular fossils” that may confer antiviral resistance without pathogenicity. Zhong et al. also reference ISH positives in ovarian and neural tissues post-IHHNV infection but note that such signals could correlate with EVE expression at viral invasion sites, reinforcing the need for caution in interpreting ISH without complementary evidence of replication. To enhance diagnostic specificity and address EVE interference, methods like the in-situ DIG-labeling loop-mediated amplification (ISDL) described by Jitrakorn et al. [32] offer promise. Targeting the IHHNV 3’ end (least prone to EVEs), ISDL outperformed conventional ISH in sensitivity [33], detecting signals in tissues like ovaries and anterior midgut cecum without proteinase-K digestion, reducing assay time to approximately 15 h vs. 3 days. It showed no cross-reactivity with other pathogens (e.g., WSSV, EHP) and could serve as a confirmatory step post-PCR/LA-PCR, providing spatial context while minimizing false positives from EVEs.

These conclusions are consistent with the cited studies [4,5], emphasizing how amplicon length influences specificity in viral detection. Sritunyalucksana et al. [2] reported that WOAH-recommended PCR methods yielded false-positive IHHNV results in 82% (9/11) of tested ponds. Despite IHHNV detection in two ponds—both co-infected with other pathogens—all ponds achieved profitable harvests. However, current practices may lead to unjustified trade rejections based solely on PCR results, which can be influenced by endogenous viral elements (EVEs).

The OIE diagnostic manual [3] cautions that conventional primers, such as 389F/R, 392F/R, and 77012F/R, can amplify both the infectious virus and EVEs, resulting in an overestimation of the prevalence of IHHNV in cultured populations. Even the 309F/R set, which is considered more specific because it does not recognize some common EVEs, is not completely free from the risk of cross-detections in certain populations.

In conclusion, at least in the case of IHHNV, where a large percentage of animals harbor endogenous viral elements (EVEs), we would like to suggest that the confirmatory test for laboratories and/or sanitary agencies like the WOAH should be histology as the primary tool for confirming the infectious nature of detections. If runt deformity syndrome (RDS) or similar characteristic traits of IHHNV are present, they should manifest as clear histological lesions, providing definitive evidence of active pathology (subclinical). While long-amplicon PCR (LA-PCR) outperforms traditional PCR by amplifying a near-complete genome and minimizing false positives from EVEs, in situ hybridization alone cannot reliably differentiate between a true replicating virus and integrated EVEs. Ultimately, the most robust approach is combining PCR methods with a sequence verification step with traditional histology to ensure accurate, comprehensive diagnostics and avoid unnecessary economic impacts in shrimp aquaculture. This recommendation is underscored by common sense and empirical evidence from the past 15 years: if IHHNV retained significant pathogenic potential or caused major pathology, Ecuador’s shrimp industry would not have achieved such remarkable production growth, becoming the world’s largest exporter of *P. vannamei* despite historical detections. Indeed, this body of evidence provides sufficient grounds to advocate for the elimination of IHHNV from the WOAH list of reportable diseases, as it no longer poses a substantial threat, allowing resources to focus on its detection and study through integrated methods as outlined above.

The World Organization for Animal Health (WOAH) maintains IHHNV as a notifiable crustacean disease due to its potential for severe economic losses, including acute mortality in *P. stylirostris* (up to 90% in juveniles) and runt deformity syndrome (RDS) that causes growth reduction in *P. vannamei* and *P. monodon*, rather than solely its subclinical effects. However, modern infections, particularly in *P. vannamei*, are often subclinical, and WOAH’s listing is primarily justified by trade risks, vertical transmission, and risks to naive wild populations, such as *P. stylirostris* [3]. Contrary to these concerns, multiple studies in the Gulf of California, a natural fishing ground for *P. stylirostris*, report no population declines or economic impacts from IHHNV in wild stocks [34,35,36,37,38]. Similarly, in *P. vannamei* aquaculture, such as in Ecuador, no significant mortality or economic losses have been attributed to IHHNV, despite high detection rates using WOAH-recommended PCR primers. One study on the Pacific coast of Latin America found no IHHNV by PCR or histopathological lesions in wild *P. stylirostris*, further challenging WOAH’s listing [24]. Reports of IHHNV affecting other species, such as *P. monodon*, often relied solely on PCR detection of IHHNV without screening for co-infections, including *Vibrio* bacteria, or conducting histology, rendering these studies incomplete [13]. Additionally, a study on hybrid shrimp, conducted over 30 years ago, reported Cowdry type A inclusions indicative of IHHNV but documented co-infections with two other viruses, complicating attribution of pathogenicity to IHHNV alone in these susceptible hybrids [11].

These findings suggest that IHHNV may be innocuous to penaeid species, such as *P. vannamei* and *P. monodon*, due to developed tolerance or, more likely, intense evolutionary pressure resulting in the integration of endogenous viral elements (EVEs), leading to persistent detection without corresponding pathogenicity.

The high sensitivity of WOAH primers may thus overestimate disease risk, leading to undue restrictions on wild shrimp fisheries and diverting resources toward managing non-pathogenic detections. We recommend adopting long-amplicon PCR (LA-PCR) and histopathology as standard diagnostic protocols to improve specificity, reduce regulatory burdens, and support sustainable shrimp fisheries. Given the lack of significant impact in modern penaeid stocks and wild populations, we further recommend that WOAH delist IHHNV as a notifiable disease to align with current evidence and promote equitable trade and resource allocation in the shrimp industry.

## 4. Materials and Methods

### 4.1. Sample Collection

Two hundred seventy-seven samples from surveillance sampling of *P. vannamei* originating from different regions in Ecuador were analyzed throughout the period spanning May 2023 and August 2025. Sampling, when possible, included animals from hatcheries, broodstock centers, farms, and wild animals. It should be noted that the shrimp sampled for PCR and histology were different individuals from the same populations. To protect client privacy, the countries or exact locations from which the samples were obtained will not be revealed here. However, the clients were informed of their responsibility to notify the competent authority of their country regarding positive test results for any shrimp pathogens listed by WOAH or arising from any unusual incidences of mortality. It would then be the responsibility of the relevant competent authorities from those member countries to report to WOAH.

### 4.2. DNA Extraction

When post-larvae (PL) were sampled, at least 100 PL were analyzed. Depending on the size of the animal or sample received, tissues were collected as follows: two pleopods per animal from a pool of at least five individuals, or a pooled sample consisting of five pleopods, five pereiopods, and five gills per animal. The tissues were homogenized, and a 0.3 g subsample was used for DNA extraction. DNA was extracted according to the manufacturer’s protocol using the Omega, Bio-Tek E.Z.N.A. tissue DNA kit (Omega, Bio-Tek Inc., Norcross, GA, USA). Briefly, each sample was minced with sterilized scissors and then ground using a microcentrifuge pestle (Thomas Scientific, Swedesboro, NJ, USA).

The processed tissue was transferred to a clean 1.5 mL Eppendorf tube (Thomas Scientific, Swedesboro, NJ, USA), to which 500 μL of tissue lysis buffer (TL) and 25 μL of Omega Bio-tek (OB) protease solution were added. The mixture was vortexed and incubated in a thermoblock (Boekel, Boekel Scientific. Feasterville, PA, USA) at 55 °C for approximately 3 h, with vortexing every 30 min. RNA was removed by adding 4 μL of RNase A (100 mg/mL), and after mixing, the sample was kept at room temperature for 2 min. The sample was then centrifuged (using a microcentrifuge ThermoFisher, Waltham, MA, USA) at 13,500× *g* rpm for 5 min, and the supernatant was carefully transferred to a new 1.5 mL Eppendorf tube. To this, 220 μL of BL buffer was added, and the mixture was vortexed and incubated at 70 °C for 10 min. Next, 220 μL of 100% ethanol was added, vortexed, and the contents were passed through a HiBind^®^ DNA Mini Column into a 2 mL collection tube. The columns were then centrifuged at 13,500× *g* rpm for 1 min, and the filtrate was discarded. Subsequently, 500 μL of HBC buffer (diluted with 100% isopropanol) was added to the column, and the sample was spun at 13,500× *g* rpm for 30 s. The filtrate was discarded, and the column was washed twice with 700 μL of DNA wash buffer diluted with 100% ethanol, and the sample was spun at 13,500× *g* rpm for 30 s. The filtrate was discarded. This step was repeated. The column was then centrifuged at 13,500× *g* rpm for 2 min to dry it out. The dried column was placed in a new nuclease-free 1.5 mL Eppendorf tube, and 100 μL of elution buffer, which was heated to 70 °C, was added to the column. The sample was allowed to sit for 2 min before being centrifuged at 13,500× *g* rpm for 1 min. This elution step was repeated. The eluted DNA was then stored at −20 °C until needed.

### 4.3. PCR Methods Used

**Figure 8:**
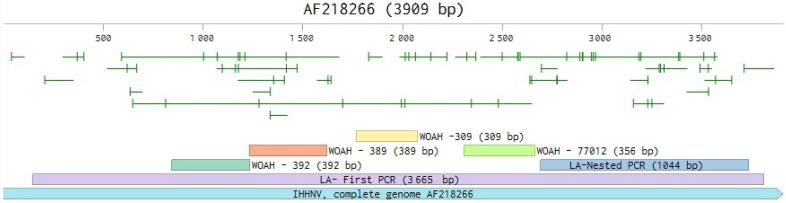
Adapted from [5], this schematic diagram illustrates the positions of the WOAH-derived primers (309 bp, 389 bp, 392 bp, and 77012F/77353R) within the full IHHNV genome (GenBank AF218266). Long-amp PCR yielded amplicons measuring 3665 bp in the first step, while nested PCR yielded amplicons measuring 1044 bp in the second.

A total of six primer sets were used to detect IHHNV using various amplification methods described in WOAH guidelines and previous publications. The first four correspond to sets IHHNV 309F/R and IHHNV 389F/R [19], IHHNV 392F/R [18], and IHHNV 77012F/77353R [21] (Figure 8). These sets amplify different regions of the viral genome, thereby increasing the sensitivity and specificity of the diagnosis. For additional verification, the IHHNV-LA (long amplification) set was applied [5] (Figure 1). This consists of a two-step nested PCR designed to amplify a high-molecular-weight fragment within the genome, thus providing robust confirmation of the virus’s presence.

PCR amplification was performed using a 20 µL PCR buffer containing 12.5 µL GoTaq^®^ Green Master Mix (Taq DNA polymerase, dNTPs, and MgCl_2_), 0.5 µL of each primer (forward and reverse), 4.5 µL ultrapure water, and 2 µL genomic DNA. Amplification was then performed in an Applied Biosystems™ ProFlex™ PCR System thermocycler under the temperature conditions described in Table 1. To visualize the results, a 2% agarose gel in 1× TBE (125 V for 30 min) was used with a 5 µL DNA intercalating dye and UV light.

### 4.4. Histopathology

For histological analysis, samples were prepared according to the protocol described by [39]. In summary, they were fixed in Davidson’s AFA solution for at least 24 h and up to 72 h in the case of broodstock. From each dissected shrimp, longitudinal sections of the cephalothorax and transverse sections of the first, third, and sixth abdominal segments were collected. Two to four paraffin blocks were prepared per specimen. Tissue sections of 5 µm thick were stained with H&E–phloxine, as described by [40]. Additionally, methyl green–pyronin staining was performed to differentiate DNA and RNA (StatLab, McKinney, TX, USA).

## 5. Conclusions

We propose delisting IHHNV from the WOAH list of reportable diseases, as neither its active form nor endogenous viral elements (EVEs) have caused significant economic impacts in any country to date. Its continued inclusion imposes unnecessary trade barriers and biosecurity restrictions, hindering global aquaculture markets and diverting research resources toward non-pathogenic detections. Given that EVEs account for most positive detections without notable pathology, we recommend standardizing long-amplicon PCR (LA-PCR) coupled with confirmatory histopathology for diagnostics in laboratories and regulatory agencies. This approach would improve diagnostic accuracy, reduce regulatory burdens, and promote sustainable aquaculture.

To address EVE-driven false positives, the FAO, WOAH, shrimp breeders, and farmer associations should collaborate to identify standardized, EVE-free target regions for pathogen detection. Such regions would reliably confirm the presence of infectious virus (positive results) or specific pathogen-free (SPF) status (negative results), enhance trade confidence, and advance sustainable practices [28]. Multiple studies in the Gulf of California, the natural habitat of *P. stylirostris*, report no population declines or economic impacts from IHHNV in wild stocks. Additionally, a study along the Pacific coast of Latin America found no evidence of IHHNV via PCR or histopathological lesions, challenging its status as a WOAH-notifiable disease [24,34,35,36,37,38]. Despite WOAH’s concerns about trade risks and vertical transmission, the listing imposes undue restrictions on wild shrimp fisheries and diverts resources toward non-pathogenic detections. We advocate adopting LA-PCR and histopathology as standard diagnostic protocols to enhance accuracy, alleviate regulatory burdens, and support sustainable shrimp fisheries

## Figures and Tables

**Table 1:** Primers and conditions used in this study for the detection of IHHNV and long-amp IHHNV.

Method	Sequences (5′–3′)	Product Lenght	Ta °C	PCR Conditions
**IHHNV 309 F/R**	(5′-TCCAACACTTAGTCAAAACCAA-3′) (5′-TGTCTGCTACG ATGATTATCCA-3′)	**309 bp**	**55**	94 °C 30 seg 35 cycles in 94 °C 30 seg, 55 °C 30 seg, 72 °C 30 seg final 72° 7 min.
**IHHNV 389 F/R**	(5′-CGG AAC ACA ACC CGA CTT TA-3′) (5′-GGC CAA GAC CAA AAT ACG AA-3′)	**389 bp**	**60**	95 °C 5 min 35 cycles in 95 °C 30 seg, 60 °C 30 seg, 72 °C 1 min final 72° 7 min.
**IHHNV 392 F/R**	(5′-GGG CGA ACC AGA ATC ACT TA-3′) (5′-ATC CGG AGG AAT CTG ATG TG-3′)	**392 bp**	**60**	
**IHHNV 77012F/77353R**	(5′-ATC-GGT-GCA-CTA-CTC-GGA-3′) (5′-TCG-TAC-TGG-CTG-TTC-ATC-3′)	**356 bp**	**60**	
**LA First** **3665 F/R**	(5′-CCCAGTTTCTAACTGACGAGTGAAGAGG-3′) (5′-CCTGACTCTAAATGACTGACTGACGATAGGG-3′)	**3665 bp**	**62**	94° 30 seg 35 cycles 94° 30 seg, 62° 30 seg, 72° 2 min 30 seg final 72° 10 min
**LA Nested** **1044 F/R**	(5′-ACAGATGTCTACAATTCAAT-3′) (5′-AATAGTAGAGAAGTGTCCC-3′)	**1044 bp**	**55**	95° 1 min 35 cycles 95° 30 seg, 55° 30 seg, 72° 1 min final 72° 5 min

## Data Availability

The datasets generated and analyzed during the current study are available in the Supplementary Materials. Due to the sensitive nature of location-specific data, access may be restricted to ensure compliance with ethical, privacy, or commercial considerations.

## Supplementary Materials

The following supporting information can be downloaded at https://www.mdpi.com/article/10.3390/ijms262311484/s1.
