# Supplementary material for: Insights on Minimizing False Positives in IHHNV Detection: Experiences from Ecuador’s Penaeus vannamei Aquaculture
Source: Int J Mol Sci. 2025 Nov 27;26(23):11484. doi: 10.3390/ijms262311484 (PMC12692355; doi:10.3390/ijms262311484)
Supplement: Supplementary file 1 [file ijms-26-11484-s001.zip › ijms-3951705-supplementary.pdf]

Table S1:

			IHHNV (OIE. 2019)					LA-PCR			
	**Matriz**	**Date**	**309 F/R (309bp)**	**389 F/R (389bp)**	**392 F/R (392bp)**	**77012F/77353R (356bp)**	**4 Primers**	**First 3665F/R**	**Nested 1044F/R**	**EVE**	**ZERO**
1	adult	06/10/2023	+	+	+	+	+			EVE	
2	adult	26/07/2023	+	+	+	+	+			EVE	
3	adult	17/07/2023	+	+	+	+	+			EVE	
4	adult	26/06/2023	+	+	+	+	+			EVE	
5	adult	21/09/2023	+	+	+	+	+			EVE	
6	adult	31/07/2023	+	+	+	+	+			EVE	
7	adult	02/05/2023	+	+	+	+	+	+	+		
8	adult	02/05/2023	+	+	+	+	+			EVE	
9	adult	17/07/2023	+	+	+	+	+	+	+		
10	adult	17/07/2023	+	+	+	+	+			EVE	
11	adult	21/09/2023	+	+	+	+	+			EVE	
12	adult	18/07/2023	+	+	+	+	+			EVE	
13	adult	02/05/2023	+	+	+	+	+			EVE	
14	adult	02/05/2023	+	+	+	+	+	+	+		
15	adult	02/05/2025	+	+	+	+	+		+		
16	adult	02/05/2025	+	+	+	+	+	+	+		
17	adult	02/05/2023	+	+	+	+	+			EVE	
18	adult	02/05/2023	+	+	+	+	+			EVE	
19	adult	08/05/2023	+	+	+	+	+			EVE	
20	adult	08/05/2023	+	+		+				EVE	
21	adult	27/09/2023	+	+	+	+	+			EVE	
22	adult	27/09/2023	+	+	+	+	+			EVE	
23	adult	27/09/2023	+	+	+	+	+			EVE	
24	adult	27/09/2023	+	+	+	+	+			EVE	
25	adult	07/07/2023	+	+	+	+	+		+		
26	adult	07/07/2023	+	+	+					EVE	
27	adult	19/07/2023	+	+						EVE	
28	adult	8/8/203	+	+		+				EVE	
29	adult	08/08/2023	+	+		+				EVE	
30	adult	19/07/2024	+	+	+	+	+			EVE	
31	adult	24/07/2024	+	+	+	+	+			EVE	
32	adult	24/07/2024	+	+	+	+	+		+		
33	adult	24/07/2024	+	+	+	+	+			EVE	
34	adult	24/07/2024	+	+	+	+	+		+		
35	adult	24/07/2024	+	+	+	+	+		+		
36	adult	24/07/2024	+	+	+	+	+		+		
37	adult	24/07/2024	+	+	+	+	+	+	+		
38	adult	24/07/2024	+	+	+	+	+	+	+		
39	adult	24/07/2024	+	+	+	+	+	+	+		

40	adult	24/07/2024	+	+	+	+	+	+	+		
41	adult	24/07/2024	+	+	+	+	+	+	+		
42	adult	24/07/2024	+	+	+	+	+	+	+		
43	adult	24/07/2024	+	+	+	+	+	+	+		
44	adult	24/07/2024	+	+	+	+	+	+	+		
45	adult	24/07/2024	+	+	+	+	+	+	+		
46	adult	24/07/2024	+	+	+	+	+	+	+		
47	adult	24/07/2024	+	+	+	+	+	+	+		
48	adult	29/07/2024	+	+	+	+	+		+		
49	adult	22/08/2024	+	+	+	+	+		+		
50	adult	22/08/2024	+	+	+	+	+	+	+		
51	adult	22/08/2024	+	+	+	+	+	+	+		
52	adult	22/08/2024	+	+	+	+	+	+	+		
53	adult	23/08/2024	+	+	+	+	+			EVE	
54	adult	23/08/2024									FREE
55	adult	23/08/2024									FREE
56	PL	23/08/2024	+	+	+	+	+			EVE	
57	Juvenile	26/08/2024									FREE
58	Juvenile	26/08/2024	+							EVE	
59	Juvenile	26/08/2024	+	+	+					EVE	
60	Juvenile	26/08/2024									FREE
61	Juvenile	26/08/2024	+	+	+					EVE	
62	Juvenile	26/08/2024	+		+					EVE	
63	Juvenile	28/08/2024	+	+	+					EVE	
64	Juvenile	28/08/2024	+	+	+	+	+			EVE	
65	Juvenile	28/08/2024	+	+	+	+	+		+		
66	Juvenile	29/08/2024		+	+				+		
67	Juvenile	29/08/2024		+	+	+				EVE	
68	Juvenile	06/09/2024	+	+	+					EVE	
69	Juvenile	06/09/2024	+	+	+	+	+			EVE	
70	Juvenile	09/09/2024	+	+	+	+	+	+	+		
71	Juvenile	09/09/2024	+	+	+	+	+		+		
72	Juvenile	09/09/2024	+	+	+	+	+	+	+		
73	Juvenile	09/09/2024	+	+	+	+	+			EVE	
74	Juvenile	11/09/2024									FREE
75	Juvenile	11/09/2024	+							EVE	
76	Juvenile	11/09/2024	+	+	+	+	+			EVE	
77	Juvenile	11/09/2024	+		+					EVE	
78	Juvenile	13/09/2024	+	+	+	+	+			EVE	
79	Juvenile	13/09/2024	+	+	+	+	+		+		
80	Juvenile	13/09/2024	+	+	+	+	+			EVE	
81	Juvenile	13/09/2024	+	+	+	+	+			EVE	
82	Juvenile	23/09/2024			+					EVE	
83	Juvenile	23/09/2024		+						EVE	
84	Juvenile	23/09/2024									FREE
85	Juvenile	26/09/2024									FREE
86	Juvenile	26/09/2024									FREE
87	Juvenile	26/09/2024									FREE
88	Juvenile	26/09/2024									FREE
89	Juvenile	26/09/2024									FREE

90	Juvenile	26/09/2024								FREE
91	Juvenile	26/09/2024								FREE
92	Juvenile	26/09/2024								FREE
93	Juvenile	26/09/2024	+	+	+				EVE	
94	adult	26/09/2024	+	+	+	+	+		EVE	
95	adult	26/09/2024	+	+	+	+	+		EVE	
96	adult	27/09/2024								FREE
97	adult	27/09/2024								FREE
98	adult	27/09/2024								FREE
99	adult	03/10/2024	+	+	+	+	+	+		
100	adult	03/10/2024								FREE
101	adult	03/10/2024	+	+	+	+	+	+		
102	adult	03/10/2024	+	+	+	+	+	+		
103	adult	03/10/2024	+	+	+	+	+	+		
104	adult	03/10/2024	+	+	+	+	+	+		
105	adult	03/10/2024	+	+	+	+	+	+		
106	adult	08/10/2024	+	+	+	+	+		EVE	
107	adult	08/10/2024	+	+	+	+	+	+		
108	adult	08/10/2024	+	+	+	+	+	+		
109	adult	08/10/2024	+	+	+	+	+	+		
110	adult	08/10/2024	+	+	+	+	+	+		
111	adult	10/10/2024								FREE
112	adult	05/11/2024	+	+	+	+	+		EVE	
113	adult	05/11/2024	+	+	+	+	+		EVE	
114	adult	05/11/2024								FREE
115	Juvenile	14/11/2024	+	+	+	+	+	+		
116	Juvenile	20/11/2024								FREE
117	Juvenile	20/11/2024								FREE
118	Juvenile	20/11/2024								FREE
119	Juvenile	21/11/2024								FREE
120	Juvenile	28/11/2024								FREE
121	Juvenile	28/11/2024	+	+	+	+	+		EVE	
122	Juvenile	28/11/2024								FREE
123	Juvenile	06/12/2024	+	+	+	+	+	+		
124	Juvenile	11/12/2024								FREE
125	Juvenile	11/12/2024								FREE
126	Juvenile	17/12/2024	+	+	+	+	+		EVE	
127	Juvenile	17/12/2024	+	+	+	+	+		EVE	
128	Juvenile	17/12/2024	+	+	+				EVE	
129	PL	17/01/2025	+	+	+	+	+	+		
130	PL	17/01/2025	+	+	+	+	+		EVE	
131	PL	17/01/2025	+	+	+	+	+		EVE	
132	PL	31/01/2025	+	+	+	+	+		EVE	

133	PL	31/01/2025	+	+	+	+	+			EVE	
134	PL	31/01/2025	+	+	+	+	+			EVE	
135	PL	21/01/2025									FREE
136	PL	21/01/2025									FREE
137	PL	30/01/2025									FREE
138	PL	30/01/2025									FREE
139	PL	30/01/2025									FREE
140	PL	30/01/2025									FREE
141	PL	30/01/2025									FREE
142	PL	11/02/2025	+	+	+	+	+			EVE	
143	PL	11/02/2025	+	+	+	+	+			EVE	
144	PL	19/02/2025	+	+	+	+	+			EVE	
145	PL	19/02/2025									FREE
146	PL	15/02/2025									FREE
147	PL	15/02/2025									FREE
148	PL	03/04/2025	+	+	+	+	+			EVE	
149	PL	03/04/2025	+	+	+	+	+	+	+		
150	PL	03/04/2025	+	+	+	+	+	+	+		
151	PL	03/04/2025	+	+	+	+	+			EVE	
152	PL	03/04/2025	+	+	+	+	+			EVE	
153	PL	03/04/2025	+	+	+	+	+			EVE	
154	PL	03/04/2025	+	+	+	+	+	+	+		
155	PL	03/04/2025	+	+	+	+	+			EVE	
156	PL	03/04/2025	+	+	+	+	+			EVE	
157	PL	03/04/2025	+							EVE	
158	PL	14/04/2025	+	+	+	+	+		+		
159	PL	14/04/2025	+	+	+	+	+			EVE	
160	PL	14/04/2025	+	+	+	+	+			EVE	
161	PL	14/04/2025	+	+	+	+	+			EVE	
162	adult	15/05/2025	+	+	+	+	+	+	+		
163	adult	15/05/2025	+	+	+	+	+	+	+		
164	adult	15/05/2025	+	+	+	+	+	+	+		
165	adult	15/05/2025	+	+	+	+	+	+	+		
166	adult	15/05/2025	+	+	+	+	+	+	+		
167	adult	15/05/2025	+	+	+	+	+	+	+		
168	adult	15/05/2025	+	+	+	+	+	+	+		
169	adult	15/05/2025	+	+	+	+	+	+	+		
170	adult	15/05/2025	+	+	+	+	+	+	+		
171	adult	15/05/2025	+	+	+	+	+	+	+		
172	adult	15/05/2025	+	+	+	+	+	+	+		
173	adult	15/05/2025	+	+	+	+	+	+	+		
174	adult	15/05/2025	+	+	+	+	+			EVE	
175	adult	15/05/2025	+	+	+	+	+	+	+		

176	adult	15/05/2025	+	+	+	+	+		+	
177	adult	21/05/2025	+	+	+	+	+			EVE
178	adult	21/05/2025	+	+	+	+	+	+	+	
179	adult	21/05/2025	+	+	+	+	+			EVE
180	adult	21/05/2025	+	+	+	+	+	+	+	
181	adult	21/05/2025	+	+	+	+	+	+	+	
182	adult	21/05/2025	+	+	+	+	+			EVE
183	adult	21/05/2025	+	+	+	+	+	+	+	
184	adult	21/05/2025	+	+	+	+	+	+	+	
185	adult	21/05/2025	+	+	+	+	+			EVE
186	adult	21/05/2025	+	+	+	+	+	+	+	
187	adult	21/05/2025	+	+	+	+	+			EVE
188	adult	21/05/2025	+	+	+	+	+			EVE
189	adult	21/05/2025	+	+	+	+	+			EVE
190	adult	21/05/2025	+	+	+	+	+			EVE
191	adult	21/05/2025	+	+		+				EVE
192	adult	21/05/2025	+	+	+	+	+	+	+	
193	adult	21/05/2025	+	+	+	+	+	+	+	
194	adult	21/05/2025	+	+	+	+	+	+	+	
195	adult	21/05/2025	+	+	+	+	+	+	+	
196	adult	21/05/2025	+	+	+	+	+	+	+	
197	adult	21/05/2025	+	+	+	+	+			EVE
198	adult	21/05/2025	+	+	+	+	+			EVE
199	adult	21/05/2025	+	+	+	+	+			EVE
200	adult	21/05/2025	+	+	+	+	+			EVE
201	adult	21/05/2025	+	+	+	+	+	+	+	
202	adult	21/05/2025	+	+	+	+	+			EVE
203	adult	21/05/2025	+	+	+	+	+			EVE
204	adult	21/05/2025	+	+	+	+	+			EVE
205	adult	21/05/2025	+	+	+	+	+			EVE
206	adult	21/05/2025	+	+	+	+	+			EVE
207	adult	21/05/2025	+	+	+	+	+			EVE
208	adult	21/05/2025	+	+	+	+	+			EVE
209	adult	21/05/2025	+	+	+	+	+			EVE
210	adult	21/05/2025	+	+	+	+	+			EVE
211	adult	21/05/2025	+	+	+	+	+			EVE
212	Juvenile	28/05/2025	+	+	+	+	+			EVE
213	Juvenile	28/05/2025	+	+	+	+	+			EVE
214	Juvenile	12/06/2025	+	+	+	+	+			EVE
215	Juvenile	12/06/2025	+	+	+	+	+			EVE
216	Juvenile	12/06/2025	+	+	+	+	+			EVE
217	Juvenile	12/06/2025	+	+	+	+	+			EVE
218	Juvenile	12/06/2025	+	+	+	+	+			EVE

219	Juvenile	12/06/2025	+	+	+	+	+		EVE	
220	Juvenile	12/06/2025	+	+	+	+	+		EVE	
221	Juvenile	12/06/2025	+	+	+	+	+		EVE	
222	Juvenile	12/06/2025	+	+	+	+	+		EVE	
223	Juvenile	12/06/2025	+	+	+	+	+		EVE	
224	Juvenile	12/06/2025	+	+	+	+	+		EVE	
225	Juvenile	12/06/2025	+	+	+	+	+		EVE	
226	adult	20/06/2025	+	+	+				EVE	
227	adult	20/06/2025	+	+	+	+	+		EVE	
228	adult	20/06/2025	+	+	+	+	+		EVE	
229	adult	20/06/2025	+	+	+	+	+	+		
230	adult	20/06/2025	+	+	+				EVE	
231	adult	20/06/2025	+	+	+				EVE	
232	adult	20/06/2025								FREE
233	adult	20/06/2025	+	+	+	+	+		EVE	
234	adult	20/06/2025	+	+	+	+	+		EVE	
235	adult	20/06/2025	+	+	+	+	+	+		
236	adult	20/06/2025	+	+	+	+	+		EVE	
237	adult	20/06/2025	+	+	+	+	+		EVE	
238	adult	20/06/2025	+	+	+	+	+		EVE	
239	adult	20/06/2025	+	+	+	+	+		EVE	
240	adult	25/06/2025								FREE
241	adult	30/06/2025	+	+	+	+	+		EVE	
242	adult	04/07/2025	+	+	+	+	+		EVE	
243	adult	04/07/2025	+	+	+				EVE	
244	adult	04/07/2025	+	+	+	+	+		EVE	
245	adult	04/07/2025	+	+	+	+	+		EVE	
246	Juvenile	03/07/2025	+	+	+				EVE	
247	Juvenile	03/07/2025	+	+	+	+	+		EVE	
248	Juvenile	03/07/2025	+	+	+			+		
249	Juvenile	03/07/2025	+	+	+				EVE	
250	Juvenile	03/07/2025	+	+	+	+	+		EVE	
251	Juvenile	03/07/2025	+	+	+				EVE	
252	Juvenile	03/07/2025								FREE
253	adult	15/07/2025	+	+	+				EVE	
254	adult	15/07/2025	+	+	+				EVE	
255	adult	15/07/2025	+	+	+				EVE	
256	adult	15/07/2025	+	+	+				EVE	
257	adult	15/07/2025	+	+	+	+	+		EVE	
258	adult	15/07/2025	+	+	+				EVE	
259	adult	15/07/2025	+	+	+	+	+		EVE	
260	adult	15/07/2025	+	+	+	+			EVE	
261	adult	15/07/2025		+	+				EVE	

262	adult	15/07/2025	+	+	+	+	+			EVE	
263	adult	00/01/1900	+	+	+					EVE	
264	adult	08/12/2025	+	+	+				+		
265	adult	08/12/2025	+	+	+					EVE	
266	adult	08/12/2025	+	+		+				EVE	
267	adult	08/12/2025	+	+	+	+	+			EVE	
268	adult	08/12/2025		+	+	+				EVE	
269	adult	08/12/2025	+	+	+	+	+			EVE	
270	adult	08/12/2025	+	+		+				EVE	
271	adult	08/12/2025	+	+	+	+	+			EVE	
272	adult	08/12/2025	+	+	+	+	+			EVE	
273	adult	08/12/2025	+	+	+	+	+		+		
274	adult	08/12/2025	+	+	+	+	+		+		
275	adult	08/12/2025	+	+					+		
276	adult	08/12/2025	+		+	+				EVE	
277	adult	08/12/2025	+	+	+	+	+			EVE	
	**Positives**		**230**	**229**	**224**	**202**	**192**		**82**	**154**	**41**
	**%**		**83.0%**	**82.7%**	**80.9%**	**72.9%**	**69.3%**		**29.6%**	**55.6%**	**14.8%**
